# Molecular Characterization of Laboratory Mutants of *Fusarium oxysporum* f. sp. *niveum* Resistant to Prothioconazole, a Demethylation Inhibitor (DMI) Fungicide

**DOI:** 10.3390/jof7090704

**Published:** 2021-08-28

**Authors:** Owen Hudson, Sumyya Waliullah, Pingsheng Ji, Md Emran Ali

**Affiliations:** Department of Plant Pathology, University of Georgia, Tifton, GA 31793, USA; Owen.hudson@uga.edu (O.H.); Sumyya.Waliullah@uga.edu (S.W.); pji@uga.edu (P.J.)

**Keywords:** fungicide resistance, *Fusarium oxysporum niveum*, Fusarium wilt of watermelon, prothioconazole, DMI fungicide, mutagenesis, gene expression

## Abstract

*Fusarium oxysporum* f. sp. *niveum* (FON) is the causal agent of Fusarium wilt in watermelon, an international growth-limiting pathogen of watermelon cultivation. A single demethylation inhibitor (DMI) fungicide, prothioconazole, is registered to control this pathogen, so the risk of resistance arising in the field is high. To determine and predict the mechanism by which FON could develop resistance to prothioconazole, FON isolates were mutagenized using UV irradiation and subsequent fungicide exposure to create artificially resistant mutants. Isolates were then put into three groups based on the EC_50_ values: sensitive, intermediately resistant, and highly resistant. The mean EC_50_ values were 4.98 µg/mL for the sensitive, 31.77 µg/mL for the intermediately resistant, and 108.33 µg/mL for the highly resistant isolates. Isolates were then sequenced and analyzed for differences in both the coding and promoter regions. Two mutations were found that conferred amino acid changes in the target gene, *CYP51A*, in both intermediately and highly resistant mutants. An expression analysis for the gene *CYP51A* also showed a significant increase in the expression of the highly resistant mutants compared to the sensitive controls. In this study, we were able to identify two potential mechanisms of resistance to the DMI fungicide prothioconazole in FON isolates: gene overexpression and multiple point mutations. This research should expedite growers’ and researchers’ ability to detect and manage fungicide-resistant phytopathogens.

## 1. Introduction

Fusarium wilt of watermelon, caused by the ascomycete fungus *Fusarium oxysporum* f. sp. *niveum* (FON), is a leading factor limiting watermelon production worldwide [1,2,3,4,5]. Symptoms include single vine wilting, tip necrosis, dieback, and eventual plant death. This widespread pathogen is soil-borne and produces three different spore types: microconidia, macroconidia, and chlamydospores [3,6]. Symptoms are caused by the host defense response to develop tyloses which attempt to block the vascular spread of the pathogen. Developing tyloses then clog up the passage of water and nutrients within the plant, causing loss of turgor pressure and wilting [7,8]. While micro- and macroconidia cause in-season spread of FON and hyphal structures can survive overwinter, chlamydospores can survive in soils for up to 10 years and are resistant to current management measures [9,10,11]. In addition to resistant spore types, FON has evolved multiple races (0, 1, 2, 3), some of which are highly aggressive on all commercial watermelon cultivars [12,13].

Management strategies have been reduced since the phasing out of methyl bromide as a soil fumigant due to its negative effect on the ozone layer [14]. Other soil fumigants have been used (chloropicrin and metam sodium), but they are not as effective as methyl bromide, so new chemistries and strategies are needed [2,3,15]. Crop rotation and nematode management have shown some success, but due to the prolonged survival of chlamydospores, these strategies have proven insufficient to halt their spread [16,17]. Apart from fumigants, a single fungicide, prothioconazole (Proline 480 SC; Bayer CropScience, Research Triangle Park, NC), is labeled for control of FON on watermelons [7]. Prothioconazole is a demethylation inhibitor (DMI) fungicide and has been tested in several studies to determine the sensitivity of FON populations [7,18,19]. To date, no reports of resistance have been made; however, management continues to be problematic [7,19]. While other fungicides are being developed to control FON on watermelon, growers’ options are limited, and reports of insensitivity do occur when talking with growers (personal communication) [19,20]. Previous studies on FON sensitivity to prothioconazole determined that 10 µg/mL inhibited growth of all isolates, though spore germination was not inhibited greatly [7,19].

DMI fungicides are under a medium risk of developing resistance; however, due to the single active ingredient registered for the pathogen, this likelihood is increased [21,22]. DMI fungicides work by inhibiting the biosynthesis of ergosterol, a crucial component of fungal plasma membranes which is required for growth and development [21]. Specifically, DMI fungicides bind to the cytochrome P450 lanosterol 14α-demethylase (*CYP51*) to inhibit ergosterol biosynthesis [23].

There are three known mechanisms of fungicide resistance for DMI fungicides, each of which has variants of the specific aberration that conveys the resistance [24]. The first mechanism is single nucleotide polymorphisms (SNPs), which alter the amino acid product and thus do not allow for proper binding of the fungicide to the gene product [25,26]. There are a number of these SNPs reported to confer resistance; some are common across multiple genera, others are specific to species or even individuals [27]. The second mechanism is overexpression of the *CYP51* gene, often due to insertions or deletions within the upstream promoter region of *CYP51* [28,29,30]. The third mechanism is increased effectivity of drug efflux transporters such as ATP binding cassette (ABC) transporter genes [31,32,33,34]. In many *Fusarium* species, three copies of *CYP51* exist: *CYP51A*, *CYP51*B, and *CYP51*C, each with a different level of activity and the ability to “cover” for a separate copy [25,27,35]. As no mechanism for resistance has been determined for FON, the objective of this study was to artificially mutate a FON isolate to become resistant to prothioconazole, then determine the mechanism by which the resistance had arisen. Greenhouse assays were included to assess the real-world impacts of the mutagenesis and resulting fungicide resistance. This study will provide a plausible mechanism for researchers to detect when resistance occurs naturally.

## 2. Materials and Methods

### 2.1. FON Isolates

Isolates of FON were obtained from commercial watermelon fields in Georgia by taking samples from infected plants and culturing them on semi-selective peptone pentachloronitrobenzene agar plates [36]. To test initial in vitro fungicide sensitivity, isolates were grown on full-strength potato dextrose agar (PDA) plates and subcultured on PDA plates amended with 10 µg/mL prothioconazole (pure product, Chem Service, West Chester, PA) (Figure S1). The value of 10 µg/mL was used to determine sensitivity since it completely inhibited growth of FON isolates from Georgia as reported previously [19]. The isolate B3-12 was chosen for mutagenesis because it was the most sensitive to the fungicide and allowed to compare the effects of resistance.

### 2.2. Generation of FON Mutants Resistant to Prothioconazole

Mycelial plugs of isolate B3-12 from a PDA plate were transferred to ½-strength potato dextrose broth (PDB) and incubated at room temperature under continuous light with shaking at 150 rpm. After 10 days, the liquid medium was filtered through a sterilized cheesecloth and spore concentration was quantified using a hemocytometer and then concentrated to 10^5^ spores/mL via centrifugation and decantation. A 100-microliter aliquot of the spore suspension was then spread on the fungicide-amended media and incubated for 5 h in the dark at 26 °C for spore germination. After 5 h, plates were taken to a sterile hood and exposed to UV light at a distance of 20 cm for 30 s before being incubated again for 7 days in the dark at 26 °C. The UV light was a germicidal far-UV producing a range of 254 nm. This was replicated in 10 separate plates, and three plates were subjected to the same treatment without UV exposure. After 7 days, UV-irradiated plates were inspected for growing colonies which were then transferred to PDA with no fungicide for another 7 days in the dark at 26 °C. These isolates were then plated on PDA with 10 µg/mL prothioconazole before being transferred to plates with increased fungicide concentrations (+5 µg/mL every subsequent week) until reaching 50 µg/mL, then repeated at 50 µg/mL for 3 weeks. Control isolates (not exposed to UV) were transferred to PDA with no fungicide each time mutants were transferred.

### 2.3. EC_50_ Value Determination for Sensitive and Resistant Isolates

After 20 weeks, resistant and sensitive isolates were plated on various concentrations of fungicide-amended PDA to determine EC_50_ values. Based on the growth results, isolates were separated into three groups to better categorize the EC_50_ values: sensitive, intermediately resistant, and highly resistant. The fungicide concentrations increased by a factor of ten, starting with 0 µg/mL, then 0.1, 1.0, 10, and finally 100 µg/mL. An additional concentration of 50 µg/mL was made for visualization of the mycelial growth inhibition but was not used in calculating EC_50_ values. After 14 days, five measurements per isolate were made from the center of the colony to the growing edge (radius), and the average length was calculated. This was conducted for nine resistant and four sensitive isolates in duplicate, and the values were again averaged for each concentration to obtain a mean value for each group. Using average EC_50_ values, the resistance factor (RF) was additionally calculated. RF values were calculated according to Lin et al. (2020) (using sensitive isolate EC_50_ mean value) and correlated to FON resistance levels, listed in Table 1 [37,38].

### 2.4. DNA and RNA Extraction

After determining significant differences in growth between sensitive and resistant isolates, isolates were grown on full-strength PDA plates for two weeks before 100 mg of mycelium was scraped from the plate and placed in a 1.5-milliliter safe-lock tube (Eppendorf Canada Ltd, Mississauga, ON, Canada). Four steel balls were added to each tube and homogenized using a FastPrep FP120 cell disruptor (Qbiogene, Carlsbad, CA, USA) for three rounds of speed 4.0 for 30 seconds. Samples were then extracted using DNeasy (DNA) and RNeasy (RNA) plant mini-kits (Qiagen, Valencia, CA, USA) according to the manufacturer’s protocol. Total DNA and RNA were quantified, and purity was estimated by measuring OD 260 nm and OD 260/280 nm using a NanoDrop spectrophotometer (NANODROP LITE, Thermo Scientific, Waltham, MA, USA).

### 2.5. Primer Design

The primers used in this study are listed in Table S1 and contain a mix of previously published primers and new primers developed for this study specifically. The three primers from Zheng et al. (2018) (FOCYP51Bpyes2-F and FoCYP51Bpyes2-R) and Zhang et al. (2006) (Fn-1 and Fn-2) used in this study overlapped with the whole genome sequences (WGS) of FON obtained from Hudson et al. (2020) (BioProject PRJNA656528) and deemed applicable for this research [35,39,40]. All novel primer sets used in concert with previously published primers were developed and used the same whole genome sequences mapped to the *Fusarium oxysporum* f. sp. *lycopersici* 4287 (FOL) reference genome (BioProject PRJNA342688) on the Integrative Genomics Viewer (Broad Institute, Cambridge, MA, USA). Each copy of the *CYP51* gene (A, B, and C) was identified from the FOL reference *CYP51* gene (XP_018249826.1) and aligned to the FON WGS. Primers were developed on the Integrated DNA Technologies Primer Quest^TM^ Tool. Downstream primers were designed to overlap upstream primers to obtain full coverage of the gene sequence. PCR primers ranged in size from 336 to 712 bp to obtain high-quality reads. The quantitative PCR (qPCR) primers used in the expression analysis were developed using the same method but for a product size of <200 bp.

### 2.6. Sequencing of Coding and Promoter Regions of CYP51

Extracted DNA was then amplified using a polymerase chain reaction (PCR) with the primer sets specific to each gene copy. PCR solutions totaled 50 µL, consisting of complete *Taq* polymerase (25 µL) (New England Biolabs, Ipswich, MA, USA), forward primer (20 µM), reverse primer (20 µM), and 2 µL of 150 ng/µL genomic DNA, and the rest was filled with PCR-grade H_2_O. Samples for the amplification of both coding and promoter regions were then added to a thermal cycler with the conditions listed in Table S1. PCR amplicons were confirmed as positive without contamination by running them on a 1% agarose gel and imaging using a UV geldoc (Analytik Jena, Upland, CA, USA). Samples were then purified using a commercial cleanup column (BioRad Laboratories, Hercules, CA, USA) and submitted to Retrogen (Retrogen, San Diego, CA, USA) for Sanger sequencing.

### 2.7. Exon and Promoter Sequence Analysis

Upon receipt of the sequencing results, fasta files were downloaded and aligned on Geneious V 11.1.5 (https://www.geneious.com) to one another. The sequences were separated into individual gene copies (*CYP51A*, -B, -C) and then aligned to the reference genome sequences of each copy, using both Bioproject PRJNA342688 (FOL) and Bioproject PRJNA656528 (FON) for alignment (Figures S2 and S3). Introns were then removed based on the alignment with the FOL reference genome gene *CYP51* (ID 28952942). Isolate sequences were then compared across each gene copy at the individual nucleotide level. Differences were identified when the sensitive (parental) isolate was compared with resistant isolates. Single nucleotide polymorphisms (SNPs) were determined to confer amino acid changes by translating the nucleotide sequence to the amino acid sequence on Geneious. Promoter sequences were submitted to this same process of alignment but no amino acid translation. Promoter sequences were sequenced until the first TATA box, 747 bp upstream from the start codon of the first exon.

### 2.8. Gene Expression Analysis

To further investigate the effects of mutagenesis, an expression analysis was performed to determine the relative expression levels of *CYP51A* in resistant and sensitive isolates of FON. For the expression analysis, total RNA extracted from fungal mycelium was converted into cDNA using the iScript^TM^ cDNA Synthesis Kit (Bio-Rad Laboratories, Hercules, CA, USA) according to the manufacturer’s instruction. A quantitative real-time PCR (qPCR) assay was performed on a BIORAD CFX connect real-time system (Bio-Rad Laboratories) in 10-microliter reactions consisting of 5 µL SsoAdvanced Universal SYBR^®^ Green Supermix (Bio-Rad Laboratories), 10 ng cDNA, 300 nM forward and reverse primers, and the rest was filled with dH_2_O. Newly developed primers specific to FON *CYP51A* were used to determine the expression of the candidate gene. Expression of FON from Zhang et al. (2006) was used as an endogenous control [40]. The recommended thermal cycling protocol for SsoAdvanced SYBR Green was used: activation/DNA denaturation at 95 °C for 30 s, denaturation at 95 °C for 10 s, and annealing/extension at 60 °C for 30 s for 40 cycles. A melt curve analysis was included: 65 to 95 °C in 0.5 °C increments, 5 s per step. Samples were run in Bio-Rad plastics and sealed with optical adhesive seals (Bio-Rad Laboratories). All assays included reverse-transcription-negative controls to check for genomic DNA contamination and no template controls to check for other contamination. Each reaction was run in technical triplicate. The 2^−ΔΔCt^ equation by Livak and Schmittgen (2001) was used to determine the relative gene expression [41]. Three isolates of each resistance level were run in triplicate and averaged for each group pertaining to resistance (highly resistant, intermediately resistant, and sensitive).

### 2.9. Statistical Analysis

Data are represented as mean ± SEM. Graphs were prepared and all data were analyzed using GraphPad Prism 8. Statistical significance was determined using the two-tailed Student’s t-test and Pearson’s R. *p* < 0.05 was considered statistically significant.

### 2.10. Molecular Modeling

Molecular models of CYP51A protein were created using SWISS-MODEL with the *CYP51* gene copy from *Aspergillus fumigatus* as the model reference [42]. Alignments of *FONCYP51A* were performed after intron removal using UniProtKB–A0A0D2Y5I9 on Geneious software to confirm the coding region as similar (Gene ID: 28952942). Zoomed-in regions highlight the point mutation impact on molecular structure as determined from sequencing data and SNP determination.

### 2.11. Greenhouse Trial

To prepare FON isolates for inoculation, the three FON isolates—B3-12 (not mutated sensitive isolate) (S), highly resistant (HR), and intermediately resistant (IR)—were grown on full-strength PDA for two weeks at 25 °C. Five mycelial plugs (5 mm in diameter) from the edge of the growing isolate colony were transferred aseptically to a 250-milliliter flask with 200 ml of ¼-strength potato dextrose broth (PDB). The liquid cultures were incubated for 2 weeks on an orbital shaker (G10 Gyrotory Shaker, New Brunswick Scientific Company, NJ, USA) at 130 rpm at room temperature (23 °C). After two weeks, the colonized liquid PDB was filtered through a sterile cheesecloth to remove mycelia and retain spores. The concentration of the spore suspension was adjusted to 1 × 10^6^ spores/mL by adding sterile distilled water (SDW).

In the greenhouse evaluation, watermelon seedlings (Sugar Baby) were grown in pots of 9 cm in diameter containing a mixture of sand:peat:vermiculite (4:1:1, *v*:*v*:*v*). Seedlings were inoculated after the first true leaf stage fully emerged by pipetting 5 mL of the conidial suspension near the base of each watermelon seedling. Half of the treatments received 20 mL of Proline at commercial concentrations 24 h before FON inoculations, and the rest of the plants received sterile water 24 h before FON inoculation. These were applied at the base of the crown where the soil meets the plant. Eighteen plants were grown per treatment (7 treatments) and sterile water was added for the negative control treatment. After inoculation, seedlings were maintained at 28 °C during the day and 20 °C in the evening with 70–80% relative humidity in the greenhouse. Disease severity was recorded after 4 weeks on a scale from 0 to 9, with a score of 0 for asymptomatic plants, 3 for plants with cotyledon lesions, 5 for plants showing slight wilting and stunting, 7 for plants with severe wilting and stunting, and 9 for dead plants. The 18 plants per treatment were evaluated in two separate independent greenhouse experiments using a randomized control block design. The treatments were as follows: 0: Negative control (NC); 1: Sensitive (S) without fungicide; 2: Highly resistant (HR) without fungicide; 3: Intermediately resistant (IR) without fungicide; 4: Sensitive (S) with Proline; 5: Highly resistant (HR) with Proline; 5: Intermediately resistant (IR) with Proline.

## 3. Results

### 3.1. EC_50_ Value and Resistance Factor Determination

**Figure 1:**
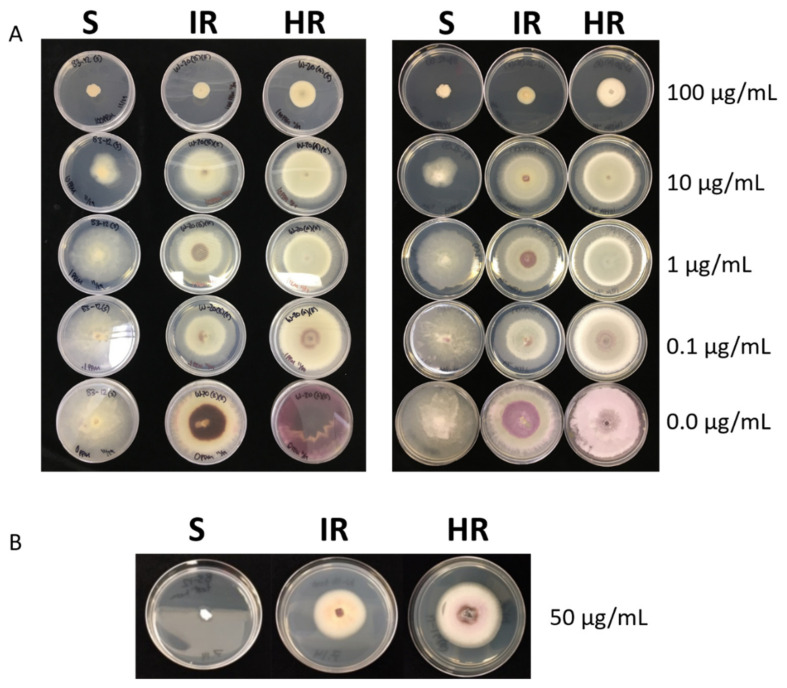
Phenotypic growth assay. (**A**) Growth assay on decreasing levels of prothioconazole-amended media from 100 to 0 µg/mL. Both sides of the media and growth are shown. S = Sensitive isolate, HR = Highly resistant isolate, IR = Intermediately resistant isolate. (**B**) The same isolates grown at 50 µg/mL prothioconazole to show the morphological differences. All plates are from 14 days post-inoculation.

In total, nine FON mutants were generated using the UV irradiation method described in the methods section that showed resistance to prothioconazole. Both resistant and sensitive parental isolates were tested to determine their EC_50_ values using a mycelial growth inhibition assay (Figure 1).

**Figure 2:**
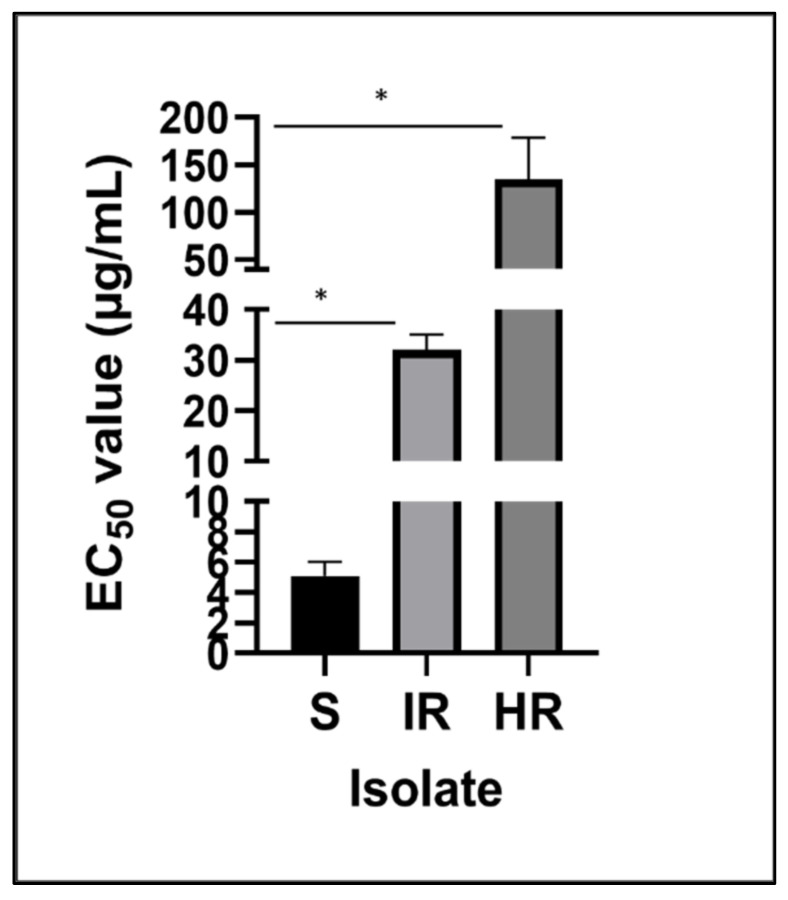
In vitro sensitivity of FON mutants to prothioconazole. Graph displays a comparison of the mean EC_50_ values among mutant isolates. Asterix (*) signifies a significant difference (α < 0.05) between the isolate group and the sensitive parent group.

For sensitive isolates, the mean EC_50_ value was 4.98 µg/mL. Resistant isolates were separated into two groups, one as intermediately resistant (IR) and the other as highly resistant (HR). Intermediately resistant isolates had a mean EC_50_ value of 31.77 µg/mL, and the highly resistant isolates had a mean EC_50_ value of 108.33 µg/mL (Figure 2).

Resistance factor (RF) values were calculated from the average EC_50_ values and determined to be 21.72 for the highly resistant isolate mean and 6.37 for the intermediately resistant isolate mean (Table S2). Unpaired two-tailed Student’s t-tests showed a significant difference between sensitive isolates and intermediately resistant (*p* = 0.001) and highly resistant (*p* = 0.042) isolates.

### 3.2. Coding Region and Promoter Sequence Analysis

**Figure 3:**
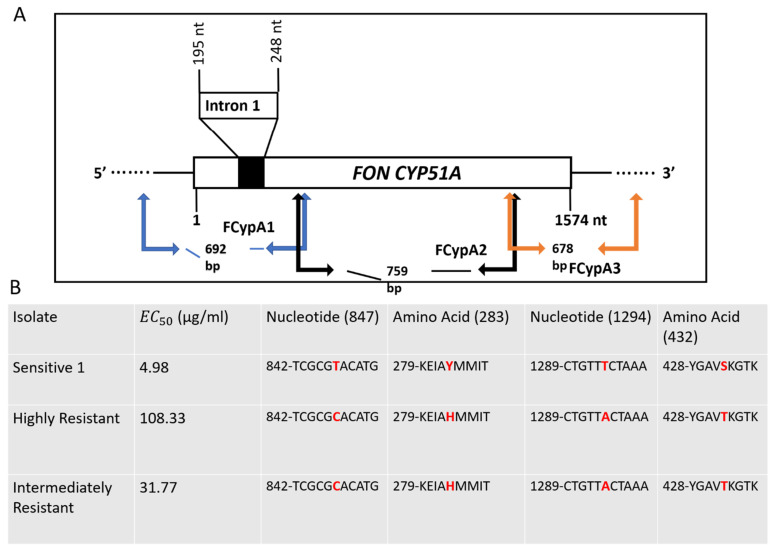
CYP51A gene schematic and detected mutations. (**A**) Intron and exon organization of *CYP51A*. Primers used for sequencing this gene are written in blue, black, and orange. (**B**) Table with isolates’ group mean EC_50_ values and mutation locations of each isolate group. Amino acids that changed from sensitive to resistant are highlighted with colored text (blue = nucleotide, red = amino acid).

CYP51A is 1574 nucleotides long in FON (a total of 524 amino acids) and contains one intron of 53 bp. Three primers (FCypA1, FCypA2, and FCypA3) successfully amplified this region (Figure 3).

**Figure 4:**
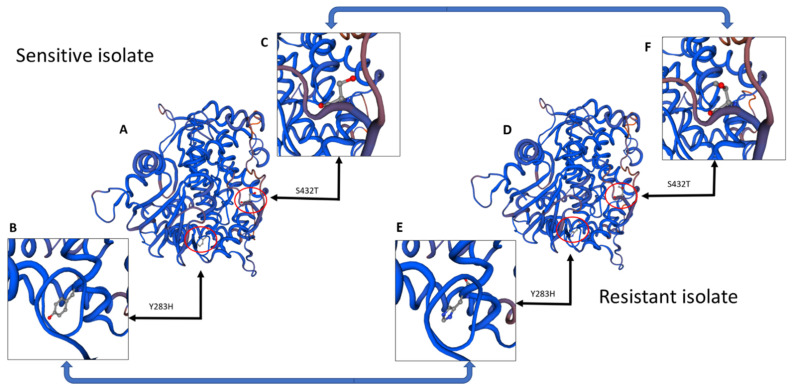
Molecular modeling of *CYP51A*. (**A**) Molecular model of fungicide-sensitive *CYP51A*, with red circles highlighting the molecular arrangement of specific amino acids mutated in the resistant version. (**B**) Zoomed-in window at tyrosine 283 to show molecular structure and location. (**C**) Zoomed-in window at serine 432 to show molecular structure and location. (**D**) Molecular model of highly fungicide-resistant *CYP51A*, with red circles highlighting the molecular arrangement of specific amino acids different from the sensitive version. (**E**) Zoomed-in window of histidine 283 (changed from tyrosine) to show molecular structure and location. (**F**) Zoomed-in window at threonine 432 (changed from serine) to show molecular structure and location.

Several mutations were seen in the coding region sequence of the resistant isolates compared to the control sensitive isolates, all of which occurred in CYP51A and none in the other two copies, CYP51B and CYP51C. In CYP51A, three point mutations occurred in the highly resistant isolate sequence and only two in the intermediately resistant isolate (Figure 3). The first mutation, at nucleotide position 847, changed from a thymine to a cytosine in both resistant isolates. This mutation conferred the amino acid change Y283H, changing a tyrosine to a histidine at amino acid position 283 (Figure 3 and Figure 4).

The second mutation occurred in only the highly resistant isolate at nucleotide position 1101 and changed an adenine to a guanine. This mutation was silent, conferring no amino acid changes. The final mutation was observed at nucleotide position 1294 in both resistant isolates and changed a thymine to an adenine, conferring the amino acid change S432T (serine to threonine) (Figure 3 and Figure 4). Three SNPs were seen in the highly resistant isolate sequence and two resulted in changes in the amino acid sequence, both of which were seen in the intermediately resistant isolate (Figure 3 and Figure 4). The promoter region sequenced was 747 bp upstream from the initial start codon to reach the first TATA box. Promoter sequences did not differ in any nucleotide across any CYP51 gene copy, and resistant isolates were identical to the sensitive parental isolate.

### 3.3. Gene Expression Analysis

**Figure 5:**
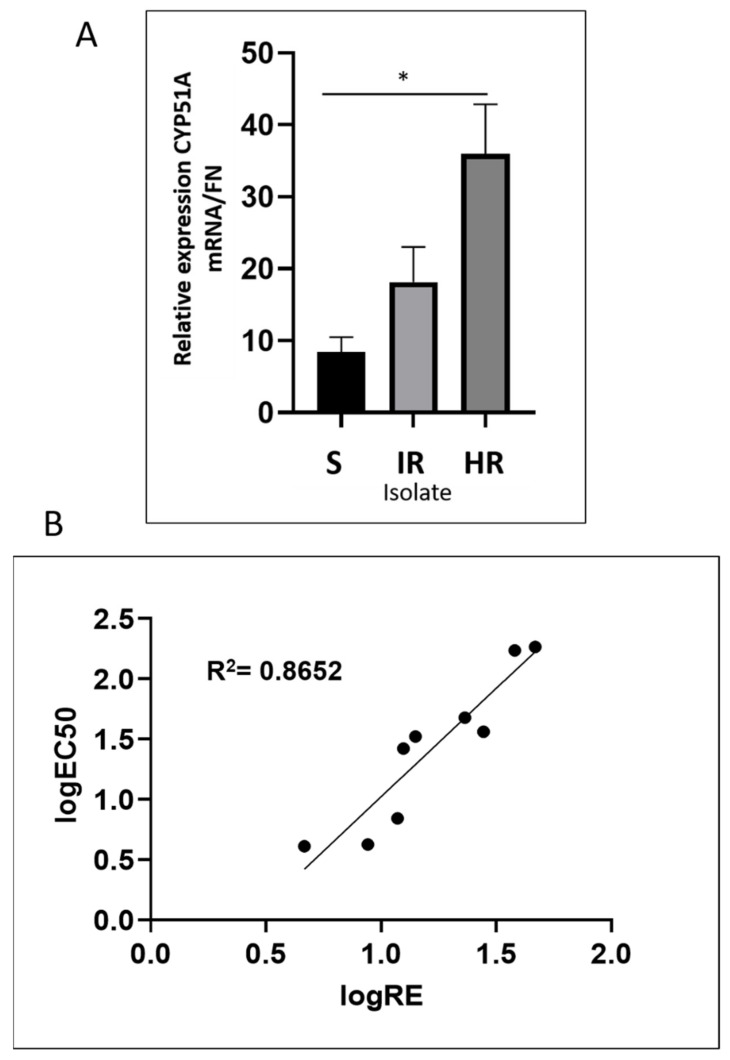
Relative expression of CYP51 and correlation with isolates’ sensitivity. (**A**) Graphical representation of the relative expression (RE) calculated with the reference FON gene using the 2^−ΔΔCt^ method. Asterix (*) indicates significant differences between sensitive and resistant groups. (**B**) Correlation between RE and EC_50_ values of *Fusarium oxysporum* f. sp. *niveum* (FON). R^2^ value = 0.8652, showing a positive and statistically significant correlation between gene expression and growth on fungicide.

Evaluation of the relative expression (RE) of the CYP51A gene among the mutants revealed that it was increased two-fold among the intermediately resistant isolates and four-fold among highly resistant isolates from the sensitive isolates (Figure 5A).

Differences in RE of CYP51A were statistically significant between both the sensitive and highly resistant isolates and the sensitive and intermediately resistant isolates. The sensitive isolate’s mean RE was 8.39, whereas the highly and intermediately resistant isolates had REs of 35.95 and 18.16, respectively. These results are 4.28 times (highly resistant) and 2.16 times (intermediately resistant) higher than that of the sensitive isolate. Log_10_ (RE) and Log_10_ (EC_50_) values were positively and significantly correlated, with an R^2^ of 0.8652 (Y = 1.8x − 0.7785) (Figure 5B).

### 3.4. Greenhouse Assay Results

Results from the greenhouse assay were averaged for each treatment across all replications (Table 2).

The treatments were then compared using several ANOVA tests comparing the means of two separate groups. First, treatments 0–3 were used to test for a difference in disease severity without the fungicide present. Second, treatments 0 and 4–6 were used to test for a difference in disease severity with the fungicide present. Finally, all treatments were analyzed using a two-tailed t-test of significance to determine whether there was a difference between the treatments receiving the spore solution and those receiving the spore solution and the fungicide (Table 3).

The ANOVA tests revealed no significant difference between the means of the two different groups. The group without the fungicide added had a *p*-value of 0.076, and the groups with the fungicide had a *p*-value of 0.673. The fungicide therefore reduced the variance in disease severity. The disease severity between the same isolates was greater without the fungicide in all cases, although t-tests revealed the highly resistant isolate replicates to have the lowest *p*-value of 0.089, which was still not statistically significant (Table 3).

## 4. Discussion

While watermelon cultivars resistant to some races of the Fusarium wilt pathogen have been developed, new races have evolved to overcome the resistance, so growers have to use other disease control methods such as chemical control. For control of *Fusarium oxysporum* f. sp. *niveum*, only prothioconazole (Proline 480 SC; Bayer CropScience, Research Triangle Park, NC, USA) is currently registered [7]. Although it is expected that other fungicides will be registered, repeated use of a single fungicide incurs significant risk of developing resistance. It is currently unknown whether FON isolates resistant to DMI fungicides (to which prothioconazole belongs) exist, but this class additionally has a medium risk of developing resistance. To better understand and predict how resistance might arise, we developed prothioconazole-resistant FON mutants that could grow well on fungicide-amended media (Figure 1).

Two resistant groups were proposed based on the EC_50_ values of prothioconazole-resistant mutants and subsequent resistant factors (RFs): intermediately resistant (IR) and highly resistant (HR) isolates. The mean EC_50_ values of the HR and IR groups compared to that of the sensitive (S) group showed resistance factors of 21.72 and 6.37, respectively (Figure 3 and Table S2). These groups were then analyzed with an unpaired two-tailed t-test for significance, revealing a significant difference between IR and sensitive (*p* = 0.001), HR and sensitive (*p* = 0.042), and HR and IR (*p* = 0.0413) (Figure 2).

Sequencing and analysis of cytochrome P450 lanosterol 14α-demethylase (*CYP51*) copies A, B, and C revealed that only *CYP51A* had mutations. While both the intermediately and highly resistant isolates had two mutations conferring amino acid changes, Y283H and S432T, the highly resistant isolate had an additional silent mutation at nucleotide position 1101 (Figure 3). Of the two mutations conferring amino acid changes, changing a tyrosine to a histidine was previously reported by Qian et al. (2017) as a mechanism for resistance of *Fusarium graminearum* to a different demethylation inhibitor (DMI), tebuconazole. Although the mutation was seen at amino acid position 137 and occurred in the *CYP51*B copy in that study, similar molecular binding alterations conferring resistance could be occurring in this study [25]. The second mutation, S432T, is not a well-characterized mutation when investigating DMI resistance, although central serine amino acids have been found to be important to molecular structure [43]. The final mutation, which was silent, occurred only in the most resistant isolate as determined by the growth assay and changed an adenine to a guanine at nucleotide position 1101. Silent mutations are not known to cause resistance to DMI fungicides; however, there is an increased presence of amino acid changes in resistant isolates of multiple phytopathogens, although often more than one [24,44,45,46]. Due to the similarities between the results in this study and the results from other studies mentioned previously, we believe it reasonable to consider these mutations to at least contribute to the fungicide resistance seen in the growth assays. Neither gene copy *CYP51*B nor *CYP51*C had any nucleotide changes in either of the resistant isolates when compared to the sensitive parental isolate. No differences were seen across the sequenced 747 bp of the promoter regions in any of the three gene copies of *CYP51* (Figures S2 and S3).

As *CYP51A* incurred mutations from the irradiation, further investigation by way of an expression analysis took place and revealed statistically significant differences between the highly resistant and sensitive isolates (Figure 5A). The RE analysis revealed that the highly resistant isolate had an expression level 2.16 times that of the intermediately resistant isolate and 4.28 times that of the sensitive parental isolate (35.95 = HR, 18.16 = IR). No mechanism was determined for the differences in expression when analyzing the promoter sequences, but it should be noted that only 747 bp of the promoter was sequenced, and additional aberrations could have occurred upstream of the first TATA box. Increases in *CYP51* gene expression have been correlated multiple times to resistance in DMI fungicides due to the increased target gene availability; thus, it is reasonable to attribute a significant level of resistance to the differences in relative expression [33,47,48].

FON greenhouse studies revealed differences in both sensitivity to the fungicide and virulence with and without the fungicide. Treatments receiving both the spore solution and the fungicide showed lower disease severity than those same treatments without the fungicide. Plants receiving the sensitive isolate spore solution were lower both with and without the fungicide, followed by the intermediately resistant isolate and, finally, the highly resistant isolate. Plants infected with sensitive isolates were almost brought down to the level of the negative control, illustrating the ability of the fungicide to reduce symptoms. Plants receiving spore solutions with the mutated isolates (HR and IR) showed a slightly higher impact (+0.27 disease severity compared with sensitive) from receiving the fungicide than the sensitive isolate but still showed higher disease severity with the resistant isolates. This implies a slightly reduced impact from the resistance detected in the morphological growth assays, which could be a result of the mutagenesis or a drawback for the pathogen to sacrifice pathogenicity for fungicide resistance. 

While definitive conclusions about the source of DMI resistance in FON populations should not be drawn from these data, the detected mutations and differences in gene expression suggest two possible mechanisms. These changes were characterized to better predict possible mechanisms of resistance to the only class of fungicides registered for FON. Further analysis of ABC transporters and other efflux transporters or expression of other gene copies should additionally be considered as they were not studied here but could be contributing to resistance. In the case of DMI resistance in FON field isolates, we hope that this research can assist in detecting the mechanism rapidly, saving resources for researchers and growers.

## Figures and Tables

**Table 1:** Sensitivity grouping based on resistant phenotype (EC_50_) values.

Resistance Factor (RF)	Resistance Level	Resistance Group
**<3.0**	Sensitive	Sensitive
**3.0–5.0**	Mild resistance	Intermediately resistant
**5.1–10.0**	Intermediate resistance	
**10.1–15.0**	Moderate resistance	
**>15.1**	High resistance	Highly resistant

**Table 2:** Watermelon seedling treatments with average disease severity.

Group	Treatments	Average Disease Severity
	0 (NC)	2.55
Group I. Absence of fungicide	1 (S)	3.61
	2 (HR)	4.83
	3 (IR)	4.27
Group II. Presence of fungicide	4 (S)	2.66
	5 (HR)	3.55
	6 (IR)	3

**Table 3:** T-test on each treatment with the same isolate across all replicates.

Treatments	*p*-Value
Treatments 1 and 4	0.204
Treatments 2 and 5	0.089
Treatments 3 and 6	0.104

## Data Availability

The data presented in this study are available within the article or supplementary material.

## Supplementary Materials

The following are available online at https://www.mdpi.com/article/10.3390/jof7090704/s1, Figure S1: Molecular models of Prothioconazole; Figure S2: Nucleotide sequence of sensitive and resistant isolates CYP51A coding region; Figure S3: Amino Acid sequence of sensitive and resistant isolates; Table S1: List of primers used in this study; Table S2: Resistant phenotype and the relative expression of the FON mutants.
