# Supplementary material for: Molecular Characterization of Laboratory Mutants of Fusarium oxysporum f. sp. niveum Resistant to Prothioconazole, a Demethylation Inhibitor (DMI) Fungicide
Source: J Fungi (Basel). 2021 Aug 28;7(9):704. doi: 10.3390/jof7090704 (PMC8466437; doi:10.3390/jof7090704)
Supplement: Supplementary file 1 [file jof-07-00704-s001.zip › jof-1338722-supplementary.pdf]

Supplemental figures and tables

Figure S1. Molecular models of Prothioconazole

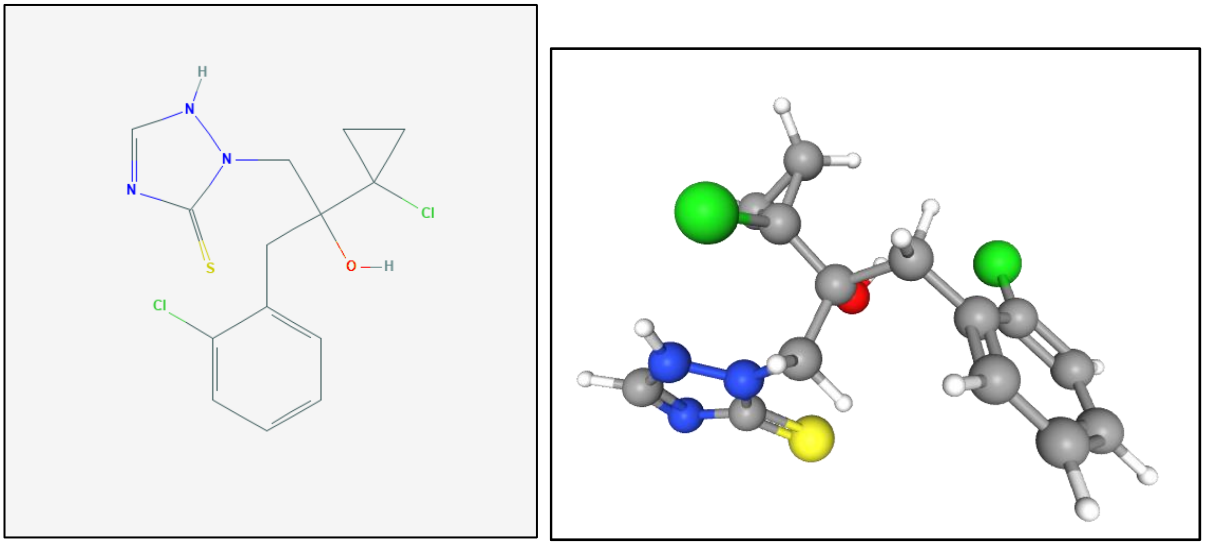

Figure S2. Nucleotide sequence of sensitive and resistant isolates CYP51A coding region

```
Sensitive1  ATGTTCTCACTCCTATACTACCCTCTATGGGCCTTTGCTTCCTGCCTAGT     50
            ||||||||||||||||||||||||||||||||||||||||||||||||||
Resistant1  ATGTTCTCACTCCTATACTACCCTCTATGGGCCTTTGCTTCCTGCCTAGT     50

Sensitive51  TATCATCACTCTCAACGTCTTATACCAGAAGCTCCCTCGAAATGCCAACG    100
             ||||||||||||||||||||||||||||||||||||||||||||||||||
Resistant51  TATCATCACTCTCAACGTCTTATACCAGAAGCTCCCTCGAAATGCCAACG    100

Sensitive101  AACCTCCGTTAGTGTTCCACTGGCTTCCATTCGTTGGGAATGCTGTTGCT    150
              ||||||||||||||||||||||||||||||||||||||||||||||||||
Resistant101  AACCTCCGTTAGTGTTCCACTGGCTTCCATTCGTTGGGAATGCTGTTGCT    150

Sensitive151  TATGGACTCGACCCTTATGGTTTCTTTGTGAAGTGTCGAGAAAAGCACGG    200
              ||||||||||||||||||||||||||||||||||||||||||||||||||
Resistant151  TATGGACTCGACCCTTATGGTTTCTTTGTGAAGTGTCGAGAAAAGCACGG    200

Sensitive201  CGATGTCTTCACCTTTATCCTCTTCGGTCGAAAAATCGTTGCCTGTCTTG    250
              ||||||||||||||||||||||||||||||||||||||||||||||||||
Resistant201  CGATGTCTTCACCTTTATCCTCTTCGGTCGAAAAATCGTTGCCTGTCTTG    250

Sensitive251  GTGTTGACGGCAATGACTTTGTTCTCAACAGTCGAATTCAGGACGCCAAC    300
              ||||||||||||||||||||||||||||||||||||||||||||||||||
Resistant251  GTGTTGACGGCAATGACTTTGTTCTCAACAGTCGAATTCAGGACGCCAAC    300

Sensitive301  GCCGAAGAAATCTACAGTCCATTGACAACGCCTGTCTTTGGTAGTGATGT    350
              ||||||||||||||||||||||||||||||||||||||||||||||||||
Resistant301  GCCGAAGAAATCTACAGTCCATTGACAACGCCTGTCTTTGGTAGTGATGT    350

Sensitive351  CGTATACGATTGTCCCAACTCGAAGCTCATGGAGCAAAAGAAGTTTGTCA    400
              ||||||||||||||||||||||||||||||||||||||||||||||||||
```

```
Resistant351 CGTATACGATTGTCCCAACTCGAAGCTCATGGAGCAAAAGAAGTTTGTCA    400

Sensitive401 AGTTTGGCCTTACACAAAAGGCTCTCGAGTCCCATGTCCAGTTGATCGAG    450
             ||||||||||||||||||||||||||||||||||||||||||||||||||
Resistant401 AGTTTGGCCTTACACAAAAGGCTCTCGAGTCCCATGTCCAGTTGATCGAG    450

Sensitive451 CGAGAGGTTCTGGAGTACATCCAAGCTGTACCTTCATTCTCTGGAAAGTC    500
             ||||||||||||||||||||||||||||||||||||||||||||||||||
Resistant451 CGAGAGGTTCTGGAGTACATCCAAGCTGTACCTTCATTCTCTGGAAAGTC    500

Sensitive501 TGGCACAGTTGATGTATCCAAGGCAATGGCTGAGATAACCATCTTCACTG    550
             ||||||||||||||||||||||||||||||||||||||||||||||||||
Resistant501 TGGCACAGTTGATGTATCCAAGGCAATGGCTGAGATAACCATCTTCACTG    550

Sensitive551 CTGCTCGCTCTCTGCAGGGCGAAGAAGTTCGACGGAAGCTTACAGCTGAG    600
             ||||||||||||||||||||||||||||||||||||||||||||||||||
Resistant551 CTGCTCGCTCTCTGCAGGGCGAAGAAGTTCGACGGAAGCTTACAGCTGAG    600

Sensitive601 TTTGCAGCTCTGTATCATGACCTTGACCTAGGCTTCACTCCTGTAAACTT    650
             ||||||||||||||||||||||||||||||||||||||||||||||||||
Resistant601 TTTGCAGCTCTGTATCATGACCTTGACCTAGGCTTCACTCCTGTAAACTT    650

Sensitive651 CCTGTTCCCTTGGCTACCTTTGCCTCATAACCGACGTCGAGATGCTGCTC    700
             ||||||||||||||||||||||||||||||||||||||||||||||||||
Resistant651 CCTGTTCCCTTGGCTACCTTTGCCTCATAACCGACGTCGAGATGCTGCTC    700

Sensitive701 ATGCAAAGATGAGAGAGATCTACATGGACATCATTAACGAACGAAGAAGA    750
             ||||||||||||||||||||||||||||||||||||||||||||||||||
Resistant701 ATGCAAAGATGAGAGAGATCTACATGGACATCATTAACGAACGAAGAAGA    750

Sensitive751 GGCGTAGGGGACTTGGAGAAAGGAACTGACATGATCGCCAACCTGATGAA    800
             ||||||||||||||||||||||||||||||||||||||||||||||||||
Resistant751 GGCGTAGGGGACTTGGAGAAAGGAACTGACATGATCGCCAACCTGATGAA    800

Sensitive801 TTGCGAGTACAAAAACGGGCAGCCGATTCCGGACAAAGAGATCGCGTACA    850
             |||||||||||||||||||||||||||||||||||||||||||||| |||
Resistant801 TTGCGAGTACAAAAACGGGCAGCCGATTCCGGACAAAGAGATCGCGCACA    850

Sensitive851 TGATGATCACTCTTCTCATGGCTGGACAACACTCTTCGTCATCTGCTAGT    900
             ||||||||||||||||||||||||||||||||||||||||||||||||||
Resistant851 TGATGATCACTCTTCTCATGGCTGGACAACACTCTTCGTCATCTGCTAGT    900

Sensitive901 TCATGGATCATACTACATCTGGCTTCATCCACTGACATTGCTGAGGAACT    950
             ||||||||||||||||||||||||||||||||||||||||||||||||||
Resistant901 TCATGGATCATACTACATCTGGCTTCATCCACTGACATTGCTGAGGAACT    950

Sensitive951 CTACCAAGAGCAACTCATTAACTTGAGTGCCGATGGTGTTCTCCCTCCCC   1000
             ||||||||||||||||||||||||||||||||||||||||||||||||||
Resistant951 CTACCAAGAGCAACTCATTAACTTGAGTGCCGATGGTGTTCTCCCTCCCC   1000

Sensitive1001 TTCAGTACTCCGATCTCGACAAGCTTCCCCTTCTTCAGAATGTCGTCAAA    1050
              ||||||||||||||||||||||||||||||||||||||||||||||||||
Resistant1001 TTCAGTACTCCGATCTCGACAAGCTTCCCCTTCTTCAGAATGTCGTCAAA    1050

Sensitive1051 GAAACACTCCGTGTTCATTCTTCCATTCACTCCATTCTGCGAAAGGTTAA    1100
              ||||||||||||||||||||||||||||||||||||||||||||||||||
Resistant1051 GAAACACTCCGTGTTCATTCTTCCATTCACTCCATTCTGCGAAAGGTTAA    1100
```

```
Sensitive1101 AAGACCTATGCAAGCAACTGGATCACCTTACACCATCACCACAGACAAGG   1150
               |||||||||||||||||||||||||||||||||||||||||||||||||
Resistant1101 GAGACCTATGCAAGCAACTGGATCACCTTACACCATCACCACAGACAAGG   1150

Sensitive1151 TTCTCCTCGCTTCACCAACTGTTACAGCGTTGAGTGAAGAACACTTCACA   1200
              ||||||||||||||||||||||||||||||||||||||||||||||||||
Resistant1151 TTCTCCTCGCTTCACCAACTGTTACAGCGTTGAGTGAAGAACACTTCACA   1200

Sensitive1201 GACGCCCAAAGATGGAATCCTCATCGGTGGGATAACAAACCCCAGGAGGA   1250
              ||||||||||||||||||||||||||||||||||||||||||||||||||
Resistant1201 GACGCCCAAAGATGGAATCCTCATCGGTGGGATAACAAACCCCAGGAGGA   1250

Sensitive1251 GGCCGTGACGGACGATGTCATTGACTACGGCTACGGCGCTGTTTCTAAAG   1300
              ||||||||||||||||||||||||||||||||||||||||||| ||||||
Resistant1251 GGCCGTGACGGACGATGTCATTGACTACGGCTACGGCGCTGTTACTAAAG   1300

Sensitive1301 GAACGAAGAGCCCATACTTACCCTTTGGCGCTGGTCGGCATCGCTGCATC   1350
              ||||||||||||||||||||||||||||||||||||||||||||||||||
Resistant1301 GAACGAAGAGCCCATACTTACCCTTTGGCGCTGGTCGGCATCGCTGCATC   1350

Sensitive1351 GGGGAGAAGTTTGCTTATGTCAACTTGGGCGTTATCGTCGCGACTTTGGT   1400
              ||||||||||||||||||||||||||||||||||||||||||||||||||
Resistant1351 GGGGAGAAGTTTGCTTATGTCAACTTGGGCGTTATCGTCGCGACTTTGGT   1400

Sensitive1401 GCGCAACTTCAGACTGTCGACTCTTGATGGCAAGCCTGGTGTTCCGGCAA   1450
              ||||||||||||||||||||||||||||||||||||||||||||||||||
Resistant1401 GCGCAACTTCAGACTGTCGACTCTTGATGGCAAGCCTGGTGTTCCGGCAA   1450

Sensitive1451 CTGACTACACTTCTCTCTTCTCAAGGCCAGCCCAACCTGCATACATAAAC   1500
              ||||||||||||||||||||||||||||||||||||||||||||||||||
Resistant1451 CTGACTACACTTCTCTCTTCTCAAGGCCAGCCCAACCTGCATACATAAAC   1500

Sensitive1501 TGGGAGCGCAGGAGGGCTTAA   1521
              |||||||||||||||||||||
Resistant1501 TGGGAGCGCAGGAGGGCTTAA   1521
```

Figure S3. Amino Acid sequence of sensitive and resistant isolates

```
Sensitive1 MFSLLYYPLWAFASCLVIITLNVLYQKLPRNANEPPLVFHWLPFVGNAVA     50
           ||||||||||||||||||||||||||||||||||||||||||||||||||
Resistant1 MFSLLYYPLWAFASCLVIITLNVLYQKLPRNANEPPLVFHWLPFVGNAVA     50

Sensitive51 YGLDPYGFFVKCREKHGDVFTFILFGRKIVACLGVDGNDFVLNSRIQDAN    100
            ||||||||||||||||||||||||||||||||||||||||||||||||||
Resistant51 YGLDPYGFFVKCREKHGDVFTFILFGRKIVACLGVDGNDFVLNSRIQDAN    100

Sensitive101 AEEIYSPLTTPVFGSDVVYDCPNSKLMEQKKFVKFGLTQKALESHVQLIE    150
             ||||||||||||||||||||||||||||||||||||||||||||||||||
Resistant101 AEEIYSPLTTPVFGSDVVYDCPNSKLMEQKKFVKFGLTQKALESHVQLIE    150

Sensitive151 REVLEYIQAVPSFSGKSGTVDVSKAMAEITIFTAARSLQGEEVRRKLTAE    200
             ||||||||||||||||||||||||||||||||||||||||||||||||||
Resistant151 REVLEYIQAVPSFSGKSGTVDVSKAMAEITIFTAARSLQGEEVRRKLTAE    200
```

```
Sensitive201 FAALYHDLDLGFTPVNFLFPWLPLPHNRRRDAAHAKMREIYMDIINERRR    250
             ||||||||||||||||||||||||||||||||||||||||||||||||||
Resistant201 FAALYHDLDLGFTPVNFLFPWLPLPHNRRRDAAHAKMREIYMDIINERRR    250

Sensitive251 GVGDLEKGTDMIANLMNCEYKNGQPIPDKEIAYMMITLLMAGQHSSSSAS    300
             |||||||||||||||||||||||||||||||| |||||||||||||||||
Resistant251 GVGDLEKGTDMIANLMNCEYKNGQPIPDKEIAHMMITLLMAGQHSSSSAS    300

Sensitive301 SWIILHLASSTDIAEELYQEQLINLSADGVLPPLQYSDLDKLPLLQNVVK    350
             ||||||||||||||||||||||||||||||||||||||||||||||||||
Resistant301 SWIILHLASSTDIAEELYQEQLINLSADGVLPPLQYSDLDKLPLLQNVVK    350

Sensitive351 ETLRVHSSIHSILRKVKRPMQATGSPYTITTDKVLLASPTVTALSEEHFT    400
             ||||||||||||||||||||||||||||||||||||||||||||||||||
Resistant351 ETLRVHSSIHSILRKVKRPMQATGSPYTITTDKVLLASPTVTALSEEHFT    400

Sensitive401 DAQRWNPHRWDNKPQEEAVTDDVIDYGYGAVSKGTKSPYLPFGAGRHRCI    450
             ||||||||||||||||||||||||||||||| ||||||||||||||||||
Resistant401 DAQRWNPHRWDNKPQEEAVTDDVIDYGYGAVTKGTKSPYLPFGAGRHRCI    450

Sensitive451 GEKFAYVNLGVIVATLVRNFRLSTLDGKPGVPATDYTSLFSRPAQPAYIN    500
             ||||||||||||||||||||||||||||||||||||||||||||||||||
Resistant451 GEKFAYVNLGVIVATLVRNFRLSTLDGKPGVPATDYTSLFSRPAQPAYIN    500

Sensitive501 WERRRA    506
             ||||||
Resistant501 WERRRA    506
```

Table S1. List of primers used in this study.

Primer name	Sequence	Assay	Conditions	expected size (bp)	Source
FCYPA1upF	GCTTACGATCGGAGAAGAACA	PCR	95 °C for 3 mins, 35 cycles of 95 °C for 30s, 58 °C for 30s, and 72 °C for 1 min, then 72 °C for 6 mins	336	This study
FCYPA1upR	AGGCCCATAGAGGGTAGTATAG				
FCYPA2upF	CCTTTGCTTCCTGCCTAGTT	PCR	95 °C for 3 mins, 35 cycles of 95 °C for 30s, 58 °C for 30s, and 72 °C for 1 min, then 72 °C for 6 mins	347	This study
FCYPA2upR	GAGCTTCGAGTTGGGACAAT				
FCYPB1upF	GTGTTTGACCGTTGTGTTTGAG	PCR	95 °C for 3 mins, 35 cycles of 95 °C for 30s, 57 °C for 30s, and 72 °C for 1 min, then 72 °C for 6 mins	364	This study
FCYPB1upR	CATGGACGGTTCCTGGAAATA				
FCYPB2upF	TATTTCCAGGAACCGTCCATG	PCR	95 °C for 3 mins, 35 cycles of 95 °C for 30s, 60 °C for 30s, and 72 °C for 1 min, then 72 °C for 6 mins	393	This study
FCYPB2upR	CGTTCCGTTTCGAAGGATGA				
FCYPC1upF	CCCAGTACATAATAGCAGGAGTG	PCR	95 °C for 3 mins, 35 cycles of 95 °C for 30s, 58 °C for 30s, and 72 °C for 1 min, then 72 °C for 6 mins	349	This study
FCYPC1upR	AAATTGGTCGCTCTGACTCAC				
FCYPC2upF	GTGAGTCAGAGCGACCAATTT	PCR	95 °C for 3 mins, 35 cycles of 95 °C for 30s, 59 °C for 30s, and 72 °C for 1 min, then 72 °C for 6 mins	408	This study
FCYPC2upR	GGTGTCGGGATGAGGATTTG				
FCypA1F	TGAGGACGCGAATCCTTCTG	PCR	95 °C for 3 mins, 35 cycles of 95 °C for 30s, 59 °C for 30s, and 72 °C for 1 min, then 72 °C for 6 mins	692	This study
FCypA1R	TGCTCCATGAGCTTCGAGTT				

FCypA2F	TCCCAACTCGAAGCTCATGG	PCR	95 °C for 3 mins, 35 cycles of 95 °C for 30s, 58 °C for 30s, and 72 °C for 1 min, then 72 °C for 6 mins	759	This study
FCypA2R	CCAGGTGCTTGCATAGGTCT				
FCypA3F	CCTATGCAAGCACCTGGATCA	PCR	95 °C for 3 mins, 35 cycles of 95 °C for 30s, 58 °C for 30s, and 72 °C for 1 min, then 72 °C for 6 mins	678	This study
FCypA3R	GTGGAATTGTGCAAATAGGGCA				
FCypA4F	TCCACCTCTACTGTTGCGAA	PCR	95 °C for 3 mins, 35 cycles of 95 °C for 30s, 58 °C for 30s, and 72 °C for 1 min, then 72 °C for 6 mins	680	This study
FCypA4R	CGACCCCGCTTATACCAAGG				
FCypA5F	CCTTGGTATAAGCGGGGTCG	PCR	95 °C for 3 mins, 35 cycles of 95 °C for 30s, 58 °C for 30s, and 72 °C for 1 min, then 72 °C for 6 mins	712	This study
FCypA5R	ACCCTACCCGCTTCTTGTTT				
FOCYP51Bpyes2-F	GGGTCTCCTCCAAGAACTT	PCR	95 °C for 3 mins, 35 cycles of 95 °C for 30s, 58 °C for 30s, and 72 °C for 1 min, then 72 °C for 6 mins	692	Zheng et al. (2018) & This study
FCypB1R	CGAACCTCGGCGGAGATAAT				
FCypB2F	CGTGCCTATTATCTCCGCCG	PCR	95 °C for 3 mins, 35 cycles of 95 °C for 30s, 58 °C for 30s, and 72 °C for 1 min, then 72 °C for 6 mins	672	This study
FCypB2R	ACAGGCATGGGAGACTTGAC				
FCypB3F	GTCAAGTCTCCCATGCCTGT	PCR	95 °C for 3 mins, 35 cycles of 95 °C for 30s, 58 °C for 30s, and 72 °C for 1 min, then 72 °C for 6 mins	444	This study & Zheng et al. (2018)
FoCYP51Bpyes2-R	CTACTGCTGGCGTCTCTC				
FCypC1F	AGCACATTCGCAACCCTGTA	PCR	95 °C for 3 mins, 35 cycles of 95 °C for 30s, 58 °C for 30s, and 72 °C for 1 min, then 72 °C for 6 mins	675	This study
FCypC1R	TTCATCACGCCGAAGCCATA				
FCypC2F	TTATGGCTTCGGCGTGATGA	PCR	95 °C for 3 mins, 35 cycles of 95 °C for 30s, 58 °C for 30s, and 72 °C for 1 min, then 72 °C for 6 mins	691	This study
FCypC2R	CCGGCAGTCCAGGTATCTTTT				
FCypC3F	AAGATACCTGGACTGCCGGA	PCR	95 °C for 3 mins, 35 cycles of 95 °C for 30s, 58 °C for 30s, and 72 °C for 1 min, then 72 °C for 6 mins	505	This study
FCypC3R	CACTGTTGGGACGCATCTA				
FCAq1F	TGGCTACCTTTGCCTCATAAC	qPCR	95 °C for 3 mins, 35 cycles of 95 °C for 30s, 58 °C for 30s, and 72 °C for 1 min, then 72 °C for 6 mins	120	This study
FCAq1R	GTCAGTTCCTTTCTCCAAGTCC				
Fn-1	TACCACTTGTTGCCTCGGC	qPCR	95°C for 2 mins, 40 cycles of 95°C for 10s and 60°C for 40s and a temperature ramp of 0.2°C/s	327	Zhang et al. (2006)
Fn-2	TTGAGGAACGCGAATTAAC				

Table S2. Resistant phenotype and the relative expression of the FON mutants

Resistant Group	EC_{50}	Resistance Factor	Relative Expression
Highly Resistant 1	47.679	9.562767	23.10286712836
Highly Resistant 2	172.228	34.5430113	38.0546276800871
Highly Resistant 3	184.78	37.0605106	46.6886510156855
HR mean	108.33	21.7272709	35.94871527
Intermediately Resistant 1	26.365	5.28791191	12.5099142900575
Intermediately Resistant 2	36.45	7.31061594	27.8576180254759

Intermediately Resistant 3	33.321	6.68304619	14.1232479406504
IR mean	31.77	6.37196895	18.16359342
Sensitive 1	4.217	0.84578511	8.75434961008591
Sensitive 2	6.969	1.39774163	11.79415374
Sensitive 3	4.079	0.81810706	4.642816
S mean	4.9859	1	8.39710634

Supplementary Table S3. Raw results from the greenhouse bioassay

Treatment	Plant 1	Plant 2	Plant 3	Plant 4	Plant 5	Plant 6	Plant 7	Plant 8	Plant 9	Plant 10	Plant 11	Plant 12	Plant 13
0 (NC)	0	0	3	3	0	3	0	0	3	3	3	7	9
1 (S)	9	5	5	3	3	3	3	0	5	3	7	5	3
2 (HR)	5	3	5	5	9	0	3	5	9	3	9	5	5
3 (IR)	3	3	5	3	3	5	0	3	3	5	9	9	7
4 (S-Pro)	7	0	0	3	0	3	0	3	3	0	3	9	5
5 (HR-Pro)	7	3	3	3	3	3	0	0	5	3	3	3	9
6 (IR-Pro)	9	3	3	3	0	0	0	3	0	0	7	7	7
0 (NC)	0	0	3	3	0	3	0	0	3	3	3	7	9

Treatment	Plant 14	Plant 15	Plant 16	Plant 17	Plant 18
0 (NC)	9	0	0	0	3
1 (S)	3	0	5	0	3
2 (HR)	7	3	3	3	5
3 (IR)	9	0	0	5	5
4 (S-Pro)	3	3	0	3	3
5 (HR-Pro)	3	5	3	3	5
6 (IR-Pro)	3	3	3	0	3
0 (NC)	9	0	0	0	3
